# Lectins from Edible Mushrooms

**DOI:** 10.3390/molecules20010446

**Published:** 2014-12-31

**Authors:** Senjam Sunil Singh, Hexiang Wang, Yau Sang Chan, Wenliang Pan, Xiuli Dan, Cui Ming Yin, Ouafae Akkouh, Tzi Bun Ng

**Affiliations:** 1 Laboratory of Protein Biochemistry, Biochemistry Department, Manipur University, Canchipur, Imphal 795003, India; E-Mail: sunil_senjam@rediffmail.com; 2 State Key Laboratory for Agrobiotechnology and Department of Microbiology, China Agricultural University, Beijing 100193, China; E-Mail: hxwang@cau.edu.cn; 3 School of Biomedical Sciences, Faculty of Medicine, The Chinese University of Hong Kong, Shatin, Hong Kong, China; E-Mails: bomberharo@yahoo.com.hk (Y.S.C.); wenliangpan@yahoo.com (W.P.); danxiuli@hotmail.com (X.D.); ycm1907@gmail.com (C.M.Y.); 4 Department of Biology and Medical Laboratory Research, Leiden University of Applied Science, Zernikedreef 11, Leiden 2333 CK, The Netherlands; E-Mail: ouafae@live.nl

**Keywords:** lectins, edible mushroom, medicinal value, biological properties, N-terminal sequences

## Abstract

Mushrooms are famous for their nutritional and medicinal values and also for the diversity of bioactive compounds they contain including lectins. The present review is an attempt to summarize and discuss data available on molecular weights, structures, biological properties, N-terminal sequences and possible applications of lectins from edible mushrooms. It further aims to update and discuss/examine the recent advancements in the study of these lectins regarding their structures, functions, and exploitable properties. A detailed tabling of all the available data for N-terminal sequences of these lectins is also presented here.

## 1. Introduction

Many species of wild mushrooms are appreciated as delicious food. They have also found a commendable place in the traditional medicines used in South-East Asian countries [1,2,3]. Edible mushrooms are the fleshy and edible fruit bodies of several species of macrofungi, which bear fruiting structures that are large enough to be seen with the naked eye. They can appear either below ground or above ground where they may be picked by hand. Edibility may be defined by criteria that include absence of poisonous effects on humans and having a desirable taste and aroma [4].

Mushrooms have been consumed by humans since ancient times, not only as a part of the normal diet but also as a delicacy (having desirable taste and aroma). In addition, the nutritional, tonic, and medicinal properties of mushrooms have been recognized for a long time. Mushrooms contain relatively large amounts of carbohydrate and fiber. Moreover, they also have a comparatively high level of protein (19%–35%, including all the essential amino acids) and are low in fat [5]. Recently, edible mushrooms have become increasingly attractive as functional foods for their potential benefit to human health [6].

Mushrooms are also known to possess a large number of nutritional, medicinal and pharmacologically important bioactive compounds including ribosome inactivating proteins, proteases, antifungal proteins, and lectins [6]. The lectins present in the mushrooms have become the subject matter of a number of studies [7,8]. So far, many mushroom lectins have been reported, and in the last few years they have attracted increased attention due to their exploitable properties encompassing a wide range of biological activities such as antiproliferative and antitumor activities toward tumor cells, hypotensive activity, immunomodulatory activity, inhibitory activity toward HIV-1 reverse transcriptase, and mitogenic activity toward spleen cells *etc.* [7,8,9,10,11].

These activities of lectins are largely attributed to the lectin–carbohydrate interactions occurring in many aspects of cellular physiology such as cell adhesion, growth and morphogenesis, molecular recognition and pathogenesis *etc.* Lectins generally interact with glycoproteins, glycolipids, and polysaccharides found on cell surfaces [10,12]. Lectins from mushrooms have also found their applications in many of the biological sciences such as in taxonomical studies, embryological and bacteriological studies, membrane glycoconjugates and cancer research, cell sorting, sorting of mutant and tumor cells and isolation of membrane and serum glyconjugates *etc.* [13].

The first study on mushroom lectins was reported in the year 1910 during toxicological investigations on *Amanita muscaria* (Fly agaric), where the lectin activity was associated with the toxicity of the mushroom. Later on, lectins from the edible mushrooms *Boletus edulis* (1912) and *Lactarius deliciosus* (1991) were reported [14]. There are many reports on lectins which were also isolated from poisonous mushrooms but their exact physiological role is still not known in detail [10]. Of these reports, mention can be made of lectins from *Chlorophyllum molybdites* [15], *Amanita phalloides* [16], *Amanita pantherina* [16], and *Inocybe umbrinella* [17], *etc.*

Mushrooms have become a major attraction for many investigators because of their exploitable biochemical constituents. It is important that these mushrooms are scientifically and thoroughly studied so that the potential use of the lectins based on their biological activities is justly tapped into. At the same time, there are lots of poisonous as well as edible mushrooms which are yet to be studied properly [18]. Recently, Singh *et al.* published an extensive review on mushroom lectins, in which 336 mushroom lectins were reported [14]. By comparing the list with edible mushrooms reported in the publication of E. Boa (2004) on wild edible fungi [19], we have identified about 144 lectins from edible mushrooms, 38 lectins from reported poisonous mushrooms, and 30 lectins from mushrooms which can be used as medicine and/or as food. Along with this, we try to highlight the available data from the literature pertaining to the structures, binding specificities and biological functions of lectins from the edible mushrooms (Table 1). These are the basic considerations which prompted us to prepare the present paper which is intended to be a review on lectins from edible mushrooms and the present status with a more updated summary of full or N-terminal amino acid sequences.

## 2. Isolation of Lectins from Edible Mushrooms

Lectins are generally carbohydrate-binding proteins found in a variety of organisms, including animals, plants, fungi, bacteria and viruses [20]. Pemberton conducted a lectin assay on more than 400 mushroom species and found that 50% of them contained lectins and many of them belonged to edible mushrooms [21]. These edible mushrooms have captured increasing attention due to their food and pharmaceutical values and also because of their bioactive components. So far, a large number of bioactive constituents have been isolated from edible mushrooms including small-molecular-weight compounds, polysaccharides, polysaccharide-protein complexes, proteins, *etc.* All these substances have interesting biological activities, such as ribosome inactivating, antimicrobial, antitumor, antioxidant, and immunomodulatory activities [22]. People started to work on edible mushroom lectins by isolating and studying their various biological activities, like those from *Tricholoma mongolicum* [23], *Tricholoma mongolicum* [24], *Volvariella volvacea* [25], *Pleurotus ostreatus* [26], *Agrocybe cylindracea* [27], *Agrocybe aegerita* [8,28], and *Pleurotus citrinopileatus* [20] *etc.*

In general, lectins from edible mushrooms are purified by using traditional purification protocols involving salt precipitation, ion-exchange chromatography, FPLC and then gel filtration steps, and sometimes along with affinity chromatography. In most of the cases, lectins from edible mushrooms were isolated from their fruiting bodies and characterized. However, there are a few reports which describe lectins from the mycelia e.g., *Tricholoma mongolicum*, *Ganoderma lucidum*, *Grifola frondosa* lectins [23,29,30]. Instances in which more than one lectin with entirely different properties were isolated from a single mushroom have also been reported [23,24,29].

**Table 1:** Structural and biological aspects of various reported lectins from edible mushrooms.

Mushroom Species	Structural Properties		Sugar Specificity	Biological Properties	References	
*Agaricus arvensis*	30.4-kDa, Homodimeric		Inulin	Antiproliferative effects against HepG2 and MCF7 tumor cells.	Zhao [31]	
*Agaricus bisporus* (ABL)	---		Galβ1,3GalNAc (TF antigen) and Sialyl Galβ1,3GalNAc	Antiproliferative effects on a range of cell types, can be useful for modulating wound healing in subconjunctival space after glaucoma surgery. Inhibits cell proliferation of some ocular and cancer cell lines	Yu [32]; Batterbury [33]; Cheung [34]	
*Agaricus blazei*	---		BSM, asialo-BSM, fetuin, asialofetuin, GalNAc	---	Kawagishi [35]	
*Agrocybe aegerita* (AAL)	15.8-kDa (AAL) Homodimeric and a member of the galectin family		Lactose BSM, glycophorin A, κ-casein, hog gastric mucin, β-galactosides, *N*-acetylglucosamine.	Tumor-suppressing function via apoptosis-inducing activity in cancer cells	Yang [8]; Yang [28]; Yang [36]; Sun [37]; Zhao [38]; Ren [39]	
*Agrocybe aegerita* (AAL2) (another novel lectin)	43-kDa, Monomeric		Non-reducing GlcNAc residues	Induces cell apoptosis *in vitro*.	Jiang [40]	
*Agrocybe cylindracea*	31.5-kDa, Heterodimeric (15.3-kDa and 16.1-kDa subunits)		Trisaccharides containing NeuAc-α2,3Galβ-(sialic acid), inulin and lactose. Also binds to simple β-galactosides, and their derivatives	Potent mitogenic activity toward mouse splenocytes	Wang [27]; Yagi [41]; Hu [42]	
*Aleuria aurantia*	72-kDa, Homodimeric Non-glycosylated, composed of two identical 312-amino acid subunits		l-Fucose and fucosyl oligosaccharides	Able to agglutinate all types of human blood erythrocytes when treated with alpha (1 leads to 2)-fucosidase.	Olausson [43]; Kochibe [44]	
*Armillaria luteo-virens*	29.4-kDa, Dimeric, fairly thermostable		Inulin	Potent mitogenic activity toward splenocytes and antiproliferative activity toward tumor cells	Feng [9]	
*Auricularia polytricha*	23-kDa, Monomeric		Raffinose, galactose, ovomucoid and β-anomers of galactoside (lactose, p-nitrophenyl-β-d-galactoside)	Able to agglutinate only trypsinized human erythrocytes.	Yagi [45]	
*Boletopsis leucomelas*	15-kDa, Monomeric		*N*-acetyl-d-glucosamine	Apoptosis-inducing activity just like mistletoe lectins	Koyama [46]	
*Boletus edulis* (BEL)	Homodimeric, 16.3-kDa subunits		d-Lactose, melibiose- and xylose-cospecific	Stimulating effect on mitogenic response of mouse splenocytes and able to inhibit HIV-1 reverse transcriptase enzyme *in vitro*. Antineoplastic or antitumor properties.	Zheng [47]; Bovi [48]; Bovi [49]	
*Boletus subtomentosus*	---		d-Lactose	---	Singh [14]	
*Clavaria purpurea* (CpL)	16-kDa, Monomeric		α-galactosyl sugar chains and raffinose	Potential interest for detection and characterization of glycoconjugates containing Galα1-4Gal and other α-galactosyl sugars on the cell surfaces.	Lyimo [50]	
*Clitocybe nebularis* (CNL)	15.9-kDa		*N,N*-diacetyllactosediamine (GalNAcb1–4GlcNAc)	Induces maturation and activation of dendritic cells via the toll-like receptor 4 pathway. Also has immunomodulatory properties on leukaemic T-cell lines. Insecticidal and anti-nutritional properties.	Svajger [51]; Pohleven [52]; Pohleven [53]	
*Coprinopsis cinerea* (CGL3)	---		Oligomers of β1-4 linked *N*-acetyl-glucosamines (chitooligosaccharides) and GalNAcβ1-4GlcNAc (LacdiNAc)	Since fungal cell walls contain chitin, CGL3 might interfere with fungal growth.	Walti [54]	
*Coprinus atramentarius*	---		d-Lactose	---	Singh [14]	
*Cordyceps militaris* (CML)	Monomeric, 31-kDa, CML comprised of 27% α-helix, 12% β-sheets, 29% β-turns, and 32% random coils		Inhibited by sialoglycoproteins	CML exhibits mitogenic activity against mouse splenocytes	Jung [7]	
*Flammulina velutipes*	12-kDa		β-d-Galactosyl residues, fetuin, human transferrin, human glycophorin, lactoferrin	Inhibits proliferation of leukemia L1210 cells	Yatohgo [55]; Ng [56]	
*Fomes fomentarius*	---		α-d-Galactosyl residues, GalNAc, raffinose	---	Singh [14]	
*Ganoderma capense*	18-kDa fairly heat stable		d(+)-galactose and d(+)-galactosamine	Potent mitogenic activity toward mouse splenocytes, and antiproliferative activity toward leukemia (L1210 and M1) cells and hepatoma (HepG2) cells	Ngai [57]	
*Ganoderma lucidum* (GLL-M and GLL-F)	GLL-M:18-kDa, GLL-F:12-kDa		M-glycoproteins (asialomucin and -fetuin). F- glucosamine and galactosamine along with glycoproteins (asialomucin, fetuin)	With health-promoting and therapeutic effects	Kawagishi [29]	
*Ganoderma lucidum* (another novel lectin from fruiting bodies)	114-kDa, hexameric lectin, lysine and tryptophan seem to be involved in sugar binding property of lectin		Glycoproteins with *N*-as well as *O*-linked glycans	---	Thakur [10,12]	
*Grifola frondosa* (GFL)	68-kDa, Homodimeric, high content of acidic and hydroxyl amino acids and low content of methionine and histidine		Terminal *N*-acetylgalactosamine-specific lectin, porcine stomach mucin, linear d-rhamnan	Cytotoxic against HeLa cells	Stepanova [30]; Kawagishi [58]	
*Gymnopilus spectabilis*	52.1-kDa and 64.4-kDa subunits Glycoprotein		Glycoproteins: fetuin, lactoferrin, and recombinant erythropoietin	Inhibits *in vitro* the growth of *Staphylococcus aureus* and *Aspergillus niger*.	Alborés [59]	
*Hericium erinaceum*	54-kDa, Heterodimeric with 15-kDa and 16-kDa subunits		Sialic acids, especially *N*-glycolylneuraminic acid	Used in Chinese medicine.	Kawagishi [60]	
*Hygrophorus russula* (HRL)	18.5-kDa Subunits (Homotetrameric)		α1-6 mannobiose isomaltose, Glcα1-6Glc	Shows mitogenic activity against spleen lymph cells (F344 rat) and strong binding of to HIV-1 gp120.	Suzuki [61]	
*Kuehneromyces mutabilis*	---		Asialo-PSM, asialofetuin, fetuin, α1 acid glycoprotein, ovomucoid	---	Singh [14]	
*Laccaria amethystine* (two lectins LALa and LALb)	LALa-17.5-kDa, Monomeric LALb-16-kDa, Monomeric		LALa–Lactose LALb—l-Fucose	---	Guillot [62]	
*Laccaria amethystine* (LAG)	17-kDa, Monomeric		Lactose and *N*-acetyllactosamine.	---	Lyimo [63]	
*Laccaria bicolor*	---		O-methylated mannose (and fucose)	Role in fungal defense against bacteria and nematodes	Wohlschlager [64]	
*Laccaria laccata*	---		l-Fucose	---	Singh [14]	
*Lactarius deliciosus* (LDL)	Dimeric, 37-kDa subunits		Specific for d-Gal β 1-3D-GalNAc residues (TF antigen)	Might play a role in the mechanism of recognition between a tree and its symbiont (fungus)	Guillot [65]	
*Lactarius deterrimus* (related)	37-kDa, Homodimeric Non-glycoprotein		Specific for [β]-d-galactosyl(1-3)-d-*N*-acetyl galactosamine residues (TF antigen)	Might play a role in recognition and specificity during the early stages of formation of mycorrhizae	Giollant [66]	
*Lactarius flavidulus*	29.8-kDa, Dimeric		lactose, p-nitrophenyl α-d-glucopyranoside, p-nitrophenyl β-d-glucopyranoside and inositol, and by the polysaccharide inulin	Suppresses the proliferation of hepatoma (HepG2) and leukemic (L1210) cells. Inhibits the activity of HIV-1RT enzyme.	Wu [67]	
*Lactarius lignyotus*	---		Asialofetuin, asialo-PSM and other desialyzed glycoproteins	---	Singh [14]	
*Lactarius rufus*	98-kDa (containing six subunits)		α-Phenyl-*N*-acetyl-d-glucosaminopyranoside, 4-nitrophenyl-β-d-Glucosamine, asialo-BSM, Human and bovine thyroglobulin	The lectin agglutinates human etrythrocytes without any marked group specificity.	Panchak [68]	
*Lactarius salmonicolor*	---		d-Gal- β1,3-d-GalNAc (TF antigen)	---	Singh [14]	
*Lactarius vellereus*	---		GalNAc	---	Singh [14]	
*Laetiporus sulfureus*	35-kDa, Hexameric Non-glycoprotein		Lactose *N*-acetyllactosamine	Hemolytic property by pore forming towards blood cells. Homologous to bacterial toxins.	Konska [69]; Mancheño [70]; Tateno [71]	
*Lentinus edodes*	43-kDa, Monomeric		Mannose, d-Melibiose, Galactosyl and glucosyl residues, *N*-acetylgalactosamine and *N*-acetylglucosamine	Mitogenic towards murine splenic lymphocytes.	Wang [72]; Moon [73]	
*Lyophyllum decastes*	10-kDa, Homodimeric		Galα1,4Gal; α-Galactosyl residues at the nonreducing terminal	The lectin shares carbohydrate binding preference with verocytoxin of bacteria *Shigella dysenteriae* and *E. coli* 0157:H7	Goldstein [74]	
*Lyophyllum shimeiji*	30-kDa		Not inhibited by simple sugars and glycoproteins.	---	Ng [75]	
*Macrolepiota procera*	16-kDa Monomeric		*N*-acetyllactosamine and other β-galactosides	Has toxic effects towards the nematode indicating a protecting role against predators and parasites	Žurga [76]	
*Marasmius oreades*	Consists of an intact (33-kDa) and truncated (23-kDa) subunit in addition to a small polypeptide (10-kDa)		Galα1,3Galβ1,4GlcNAc trisaccharide sequence; Blood group B trisaccharide (Galα1,3Gal2,1αFuc)	Human blood group B-specific lectin. Has proteolytic activity and inhibits protein and DNA synthesis in NIH/3T3 cells. May induce BAX-mediated apoptosis.	Winter [77]; Cordara [78]; Cordara [79]	
*Marasmius oreades* (MOL) (another novel lectin)	13-kDa		Mannose and thyroglobulin	---	Shimokawa [80]	
*Mycoleptodonoides aitchisonii*	64-kDa subunit, Homotetrameric		Asialo-BSM, BSM	---	Kawagishi [81]	
*Panus conchatus*	---		d-Galactose	---	Singh [14]	
*Paxillus involutus*	---		Asialo-PSM, Asialofetuin, Fetuin, α1 acid glycoprotein	---	Singh [14]	
*Pholiota adiposa*	32-kDa, Homodimeric		Inulin	Antiproliferative activity toward hepatoma Hep G2 cells and breast cancer MCF7 cells. It also exhibits HIV-1 reverse transcriptase inhibitory activity.	Zhang [82]	
*Pholiota aurivella*	---		Fetuin and Asialofetuin	---	Singh [14]	
*Pholiota squarrosa* (PhoSL)	4.5-kDa		l-Fucose α1-6-fucosylated *N*-glycans.	Able to differentiate between primary and metastatic colon cancer tissues in the expression of α1-6 fucosylation.	Singh [14]; Kobayashi [83]	
*Pleurocybella porrigens*	56-kDa, Homotetrameric		GalNAc and O-linked glycans	---	Suzuki [84]	
*Pleurotus citrinopileatus*	32.4-kDa subunits, Homodimeric		Maltose, *O*-nitrophenyl-β-d-galactopyranoside, *O/P*-nitrophenyl-β-d-glucuronide and inulin	Potent antitumor, mitogenic and HIV-1 reverse transcriptase inhibitory activities	Li [20]	
*Pleurotus ferulae*	35-kDa, Homodimeric		d-glucose, lactose, d-galactose, and galactosamine	Highly potent hemagglutinating and proliferative activities toward mouse splenocytes	Xu [85]	
*Pleurotus ostreatus.*	40- and 41-kDa subunits, Heterodimeric		Melibiose, lactose, d-galactose, a-methyl-d-galactopyranoside, *N*-acetylneuraminic acid, raffinose, and inulin. Melibiose is the most potent inhibitory sugar	Potent antitumor activity in sarcoma S-180 bearing and hepatoma H-22 bearing mice. Enhances immunogenicity of some vaccines in transgenic mice. Possesses anti-inflammatory activities	Wang [26]; Gao [86]; Jedinak [87]	
**Pleurotus* tuber-regium*	32-kDa		*N*-acetylglucosamine-binding	Exhibits hemagglutinating activity toward trypsinized rabbit erythrocytes but not toward untrypsinized rabbit erythrocytes.	Wang [88]	
*Polyporus adusta*	12-kDa subunits, Homodimeric		Turanose is the most potent inhibitory sugar	Antiproliferative activity toward tumor cell lines and mitogenic activity toward splenocytes	Wang [89]	
*Polyporus squamosus*	28-kDa subunits, Homodimeric		NeuNAcα2,6βgalactosyl residues	Can be a valuable tool for glycobiological studies in biomedical and cancer research	Mo [90]	
*Psathyrella velutina*	40-kDa, Monomeric having a regular seven-bladed β -propeller fold		*N*-acetylglucosamine and *N*-acetylneuraminic acid specific	Used in detection of glycosylation abnormality in rheumatoid IgG	Cioci [91]; Kochibe [92]	
*Russula delica*	60-kDa, Homodimeric		Inulin and *O*-nitrophenyl-beta-d-galactopyranoside	Potent inhibitor for proliferation of HepG2 hepatoma and MCF 7 breast cancer cells, also inhibits HIV-1 reverse transcriptase activity.	Zhao [93]	
*Russula lepida* (RLL)	16-kDa subunits, Homodimeric		Inulin and *O*-nitrophenyl-b-d-galacto-pyranoside	Antiproliferative activity towards hepatoma Hep G2 cells and human breast cancer MCF-7 cells	Zhang [11]	
*Russula nigricans*	---		Asialofetuin, asialo-PSM, Fetuin, Ovomucoid, α1 Acid glycoprotein	---	Singh [14]	
*Schizophyllum commune*	64-kDa, Homodimeric		Lactose-specific	Potent mitogenic activity toward mouse splenocytes, antiproliferative activity toward tumor cell lines, and inhibitory activity toward HIV-1 reverse transcriptase	Han [94]	
*Stropharia rugosoannulata* (SRL)	38-kDa, Homodimeric		Inulin	Exhibits anti-proliferative activity toward both hepatoma Hep G2, cells and leukemia L1210 cells, along with anti HIV-1 reverse transcriptase activity.	Zhang [95]	
*Tricholoma mongolicum* TML-1 and TML-2 *	37-kDa, Homodimeric, non-glycoprotein in nature	Lactose		Exhibits antiproliferative activities against mouse monocyte-macrophage PU5-1.8 cells and mouse mastocytoma P815 cells *in vitro*. Stimulates production of nitrite ions by macrophages in normal and tumor-bearing mice.	Wang [23]; Wang [24]
*Volvariella volvacea* (VVL)	32-kDa, Homodimeric, Non- glycoprotein	Inhibited not by simple sugars but by thyroglobulin		Potent stimulatory activity towards murine splenic lymphocytes showing immuno-modulatory activity. Also found to enhance transcriptional expression of interleukin-2 and interferon-γ	She [25]
*Xerocomus chrysenteron*	15-kDa	Asialofetuin, asialo-PSM and other desialyzed glycoproteins GalNAc and Gal TF antigen		It possesses a high insecticidal activity against the dipteran *Drosophila melanogaster* and the hemipteran, *Acyrthosiphon pisum*.	Trigueros [96]
*Xerocomus spadiceus*	32.2-kDa (16-kDa subunits), Dimeric	Inulin-specific		Capable of eliciting an approximately four-fold stimulation of mitogenic response in murine splenocytes	Liu [97]
*Xylaria hypoxylon*	28.8-kDa, Homodimeric	Inulin- and xylose- specific		Potent hemagglutinating activity, antiproliferative activity towards tumor cell lines, and anti-mitogenic activity on mouse splenocytes	Liu [98]

--- Data not available from the authors; * These two lectins differ in the amino acid composition (proline and tyrosine residues) and TML-2 possesses a higher hemagglutinating activity than TML-1, whereas TML-1 has a more potent antiproliferative activity against PU5-1.8 cells than TML-2, especially in the presence of serum. TML-1 possesses hypotensive and vasorelaxing action in rats [99].

## 3. Structural Properties and Sugar Specificities of Edible Mushroom Lectins

In general, lectins possess many shallow binding pockets/cassettes that are hydrophilic and the interaction with carbohydrates and lectins are typically weak. In order to achieve strength and specificity, many lectins exist as oligomers consisting of several similar or identical monomers each of which binds to the same type of carbohydrate. In this way, lectins may participate in multivalent binding, or the formation of several simultaneous binding events that provide an apparent binding affinity (functional affinity) greater than the sum of the individual interactions [10,12]. To support the aforesaid statements, Yang *et al.*, illustrated the structural basis for the apoptosis-inducing activity of an antitumor lectin from the edible mushroom *Agrocybe aegerita* (AAL) [36]. AAL has a dimeric organization and the authors showed that this dimerization of AAL is essential for its apoptosis-inducing activity toward tumor cells. Two sugars, glucose and galactose, are basic moieties of functional carbohydrate ligands for lectin bioactivity. They also identified another hydrophobic pocket essential for apoptosis-inducing activity of the lectin but which is independent of its carbohydrate binding and dimer formation. All these findings reveal a structural basis for the antitumor activities of AAL, which may even lead to the design of antitumor drugs based on the AAL prototype model [36].

Some mushroom lectins have been reported to be sensitive to inhibition of agglutination by more than a single sugar and/or sugar derivative, like lectins from *Agrocybe cylindracea* (lactose, sialic acid and inulin), *Boletus edulis* (melibiose- and xylose-cospecific), *Ganoderma capense* (d+-galactose and d+-galactosamine), *Ganoderma lucidum* (mycelial lectin inhibited by glycoproteins asialomucin and –fetuin; fruiting body lectin inhibited by glucosamine and galactosamine along with glycoproteins asialomucin, fetuin), *Lentinus edodes* (*N*-acetylgalactosamine and *N*-acetylglucosamine), *Pleurotus citrinopileatus* (maltose, *O*-nitrophenyl-β-d-galactopyranoside, *O/P*-nitrophenyl-β-d-glucuronide and inulin), *Pleurotus ostreatus* (melibiose, lactose, d-galactose, a-methyl-d-galactopyranoside, *N*-acetylneuraminic acid, raffinose, and inulin, with melibiose being the most potent), *Psathyrella velutina* (*N*-acetylglucosamine and *N*-acetylneuraminic acid specific), *Russula lepida* (inulin and *O*-nitrophenyl-β-d-galacto-pyranoside), and *Xylaria hypoxylon* (inulin- and xylose- specific) *etc.* (Table 1). Among the different sugars one of them might be the most potent inhibitory sugar. Some edible mushroom lectins have specificity towards complex carbohydrates rather than simple sugars e.g., lectins from *Volvariella volvacea*, *Ganoderma lucidum*, and *Cordyceps militaris* (Table 1). There are also some lectins whose hemagglutinating activities are unaffected by simple sugars or sugar derivatives or complex glycoproteins. Examples in this category include lectins from *Lyophyllum shimeiji*, *Agaricus edulis*, *Flammulina velutipes* and *Volvariella volvacea* [75].

The molecular weights of most lectins isolated from edible mushrooms range from 12-kDa to 68-kDa. Some lectins isolated from edible mushrooms are non-glycoproteins such as lectins from *Tricholoma mongolicum* [23], *Laetiporus sulfueus* [69], *Lactarius deterrimus* [66], and *Volvariella volvacea* [25]. Another prominent characteristic is that except for a few lectins which are either monomeric or multimeric, almost all other lectins from edible mushrooms are dimeric (Table 1). Wang *et al.* isolated two distinct lectins from the edible mushroom *Tricholoma mongolicum*, having significant biological properties [23]. These lectins (TML-1 and TML-2) have a molecular weight of 37-kDa and are non-glycoprotein in nature. The hemagglutinating activities of both lectins are sensitive to inhibition by lactose. However, it was reported that the amino acid compositions of these two lectins differ in the content of proline and tyrosine residues [99].

A structurally novel lectin (VVL) was isolated by She *et al.*, [25], from the edible straw mushroom *Volvariella volvacea*. This lectin is homodimeric with a molecular weight of 32-kDa (non-glycoprotein), and its hemagglutination activity is inhibited not by simple sugars but by thyroglobulin. Wang *et al.*, isolated a heterodimeric lectin (composed of 40-kDa and 41-kDa subunits) from fresh fruiting bodies of the edible mushroom *Pleurotus ostreatus* [26]. The inhibition of hemagglutination induced by this lectin is sensitive to inhibition by salts such as CaCl_2_, MgCl_2_, MnCL_2_ and FeCl_3_ and by sugars and sugar derivatives such as melibiose, lactose, d-galactose, α-methyl-d-galactopyranoside, *N*-acetylneuraminic acid, raffinose, and inulin. Among these sugars, melibiose is the most potent [26]. A lectin having a unique N-terminal amino acid sequence (Table 2) was isolated by Li *et al.* from an edible mushroom *Pleurotus citrinopileatus* [20]. This lectin is a homodimer with a molecular weight of 32.4-kDa and likewise this lectin is not inhibited by a single sugar. Rather, it has multiple sugar specificities (maltose, *O*-nitrophenyl-β-d-galactopyranoside, O/P-nitrophenyl-β-d-glucuronide and inulin).

**Table 2:** N-terminal amino acid sequences of lectins from edible mushrooms.

Mushroom Species	N-Terminal Sequences	Reference
*Agaricus arvensis*	TYAVLNFVYG	Zhao [100]
*Agaricus bisporus*	MGGSGTSGSL	Zhang [82]; Crenshaw [101]
*Agrocybe aegerita*	QGVNIYNI	Yang [32]; Zhao [38]
*Agrocybe cylindracea* (15.3-kDa subunit)	AVNFYNVLAGAENDLVADVE	Wang [27]
*Agrocybe cylindracea* (16.1-kDa subunit)	RVTNVANGFVAGDQKAMVRV	Wang [27]
*Coprinopsis cinerea*	IPLEGTFGDR	Walti [50]
*Flammulina velutipes*	TSLTFQLAYL	Zhang [82]; Ko [102]
*Ganoderma capense*	VNDYEAWYGADD	Ngai [57]
*Ganoderma lucidum*	QFIYNGKFNWLNYALNETIT	Thakur [10,12]
*Grifola frondosa*	NWPAEMMIDLKHPIVEMR	Kawagishi [58]
*Hericium erinaceum*	AFGQLSFANLAAADF	Li [103]
*Laccaria bicolor*	SHLYGDGVAL	Martin [104]
*Lyophyllum shimeiji*	PVVFELKFPNNNPESLLALAACARNKAH	Ng [75]
*Marasmius oreades*	YILDGEYLVL	Kruger [105]
*Paxillus involutus*	CTCAVFLNNTTVKS	Wang [106]
*Pholiota adiposa*	DILMGTYGML	Zhang [82]
*Pholiota aurivella*	YSVTTPNSVKGGTNQG	Zhang [11]; Kawagishi [107]
*Pleurotus citrinopileatus*	QYSQMAQVME	Li [20]
*Pleurotus cornucopiae*	SDSTWTFAML	Oguri [108]
*Pleurotus ostreatus* (40-kDa subunit)	ATAKIKATPAQPQQFQPAALNAAK	Wang [26]
*Pleurotus ostreatus* (41-kDa subunit)	ACATAKCTTATPQQPGCAPAALNAAK	Wang [26]
*Pleurotus* tuber-regium	DRXAGYVLYXXVPY	Wang [88]
*Russula lepida*	VWYIVAIKTDVPRTT	Zhang [11]
*Stropharia rugosoannulata*	IKSGVYRIVSWQGALGPEAR	Zhang [95]
*Volvariella* *volvacea*	PSNGNQYLIAQAYNLQKVNFDYTPQWQRGN	She [25]
*Xerocomus spadiceus*	CSKGGVGRGYGIG	Liu [97]

In one of the structural studies on lectin from *Aleuria aurantia* (AAL) using two forms of recombinant AAL produced by using site-directed mutagenesis, it was revealed that this lectin is composed of two subunits with a six-fold β-propeller structure containing five binding sites for l-fucose. The interesting finding was that all five binding sites have different binding affinities for fucose. Sites 2 and 4 have the highest affinities toward fucose, while site 1 has an intermediate affinity, and sites 3 and 5 bind fucose with weaker affinities [43].

Structural characterization of *Laetiporus sulphureus* lectins by Mancheño *et al.*, [70] shows that this lectin is a hexameric protein (composed of 35-kDa subunits) while previously it was considered as tetrameric [69,71]. It has also been established to have two distinct modules: an N-terminal lectin module and a pore-forming module. The lectin module has a β-trefoil scaffold structure resembling that of the toxins abrin and ricin. While the other module exhibits three-dimensional structural similarities with that of the bacterial β-pore-forming toxin aerolysin and є-toxin. The crystal structure of the sugar-lectin complex reveals the presence of two sugar binding sites per subunit [70].

Žurga *et al.* determined the crystal structure of the dimeric *Macrolepiota procera* lectin (MpL) and it was found that it has a β-trefoil scaffold structure. It has a carbohydrate-binding site at the α-repeat which manifests the highest specificity for terminal *N*-acetyllactosamine and other β-galactosides. Another low-affinity putative carbohydrate-binding site is also present at the γ-repeat. A second putative carbohydrate-binding site with a low affinity for galactose is present at the γ-repeat. In addition, a novel hydrophobic binding site has been detected in MpL with specificity for molecules other than carbohydrates [76].

Nowadays, lectin microarray studies have become popular as mushroom lectins are considered potent therapeutic agents. An example is the use of a high throughput miniaturized platform of lectin microarray for the detection of terminal or interior glucose, mannose and fucose residues [109]. Such structural characterizations of proteins (edible mushroom lectins) are very useful for defining their valences, specificities, and affinities. As such, they will surely have a commendable place in developing reliable diagnostic and biological assays for carbohydrate analysis.

## 4. Functional Properties of Lectins from Edible Mushrooms

Thakur *et al.*, reported that, compared to higher plants, the role of lectins in fungi seems to be more complicated [10,12]. For instance, in higher fungi (mushrooms), lectins play different roles in different situations. Some of these include functions in fungal metabolism, and other roles in symbiotic or parasitic relationships with other organisms. In addition, various roles such as parasitic and predatory behaviors have also been ascribed to the lectins in lower fungi [110]. Ng reported that mushroom lectins are localized on the caps, stipes and the mycelia [13]. These lectins may also play various crucial roles in physiological processes such as dormancy, growth, morphogenesis, morphological changes and molecular recognition during the early stages of mycorrhization [110].

Khan *et al.* also commented on the possible properties of mushroom lectins attributed to their biological roles in the host organism [111]. The ability of lectins to recognize different glycosylated structures at the levels of cells, tissues and the whole organisms endow these molecules with a number of physiological roles such as participation of parasitic mushrooms in the host organism e.g., by recognition between a tree and its symbiont (mushroom) [65]. They also help in recognition and specificity during the early stages of mycorrhizal formation, introducing morphological changes in the host and in dormancy [66]. A mechanism of how lectin plays an innate role in the host defense mechanism was also recently demonstrated by a lectin from the edible mushroom *Laccaria bicolor* (Lb-Tec2) [64]. Here, lectins not only act as recognition molecules for pathogens, they can also perform the direct defense effectors’ function by intoxicating the antagonist upon binding.

The lectin family, particularly lectins from mushrooms, has drawn growing interest from the scientific community in the last decade due to its potential importance in cancer research. Lectins from edible mushrooms such as those of *Agaricus bisporus*, *Boletus satanus*, *Flammulina velutipes*, *Ganoderma* *lucidum*, *Grifola frondosa*, *Tricholoma mongolicum*, and *Volvariella volvacea* are reported to have immunomodulatory and/or antitumor/cytotoxic/antiproliferative activities [36,57].

In 1984, during an early part of mushroom lectin research, isolation of a heterodimeric lectin from the edible mushroom *Volvariella volvacea* encouraged the search for potential agents in lectins from mushrooms for cancer therapy. This lectin has a moderate inhibitory effect on the growth of tumor cells [112]. A Gal β-1,3-GalNAc-specific lectin from the edible mushroom *Agaricus bisporus* (ABL) purified by Yu *et al.* has reversible noncytotoxic inhibiting effects on epithelial cell proliferation [32]. An *N*-acetylgalactosamine-specific lectin (GFL) from *Grifola frondosa* (GFL) fruiting bodies shows cytotoxic activity against HeLa cells. However, if this lectin has been pre-incubated with haptenic sugar *N*-acetylgalactosamine, it is unable to exhibit its cytotoxicity. This finding indicates that the sugar binding site plays an important role in providing it with its toxic effects [71].

Lectins isolated from the mushroom *Tricholoma mongolicum* (TML1 and TML2) exhibited antiproliferative activity against mouse monocyte-macrophage PU5-1.8 cells and mouse mastocytoma P815 cells *in vitro*. The same lectins also stimulated the production of nitrite ions by macrophages in normal and tumor-bearing mice and inhibited the growth of sarcoma 180 cells in the peritoneal cavity in mice [23,24]. Another lectin from *Pleurotus ostreatus* exerted potent antitumor activity in mice bearing sarcoma S-180 and hepatoma H-22 [26], while lectin from *Agrocybe cylindracea* exhibited potent mitogenic activity toward mouse splenocytes [27].

Koyama *et al.*, isolated a comparatively smaller monomeric lectin (15-kDa) from another edible mushroom Kurokawa (*Boletopsis leucomelas*), having apoptosis-inducing activity just like that of mistletoe lectins [46]. This lectin induces all the features of apoptosis such as formation of apoptotic bodies, chromatin condensation, and DNA ladder formation [46]. Another smaller (18-kDa) but fairly heat stable (0–100 °C) lectin isolated by Ngai *et al.* from the mycelial extract of mushroom *Ganoderma capense* exhibits a more potent mitogenic activity toward mouse splenocytes and antiproliferative activity toward leukemia (L1210 and M1) cells and hepatoma (HepG2) cells compared to concanavalin A [57]. Yang *et al.*, reported a member of the galectin family of lectins from the mushroom *Agrocybe aegerita* (AAL) and recombinant *Agrocybe aegerita* lectin (rAAL) having tumor cell apoptosis-inducing activity on human and mouse tumour cells [8,113]. *Pleurotus citrinopileatus* lectin is a structurally novel lectin with potent antitumor, mitogenic and HIV-1 reverse transcriptase inhibitory activities [20].

Other promising lectins include mannose-specific lectin from the mushroom *Hygrophorus russula* (HRL) which shows mitogenic activity towards spleen lymph cells of F344 rats and strong binding affinity for HIV-1 gp120 [61]. Lectin from *Lactarius flavidulus* is able to suppress the proliferation of hepatoma (HepG2) and leukemic (L1210) cells and inhibit the activity of HIV-1 RT enzyme [67]. Similarly, lectin from an edible mushroom *Pholiota squarrosa* (PhoSL) also demonstrates the ability to differentiate between primary and metastatic colon cancer tissues with regard to the expression of α1-6 fucosylation [83]. This suggests the potential application of this PhoSL as a cancer biomarker. Another novel lectin isolated from the edible mushroom *Clitocybe nebularis* (CNL) exhibits an immunostimulatory effect on most potent antigen-presenting cells, the dendritic cells (DCs) [51]. In the same paper, the authors have also shown that DCs activation by CNL is completely dependent on the toll-like receptor 4 (TLR4) activation pathway.

## 5. N-Terminal Sequences of Lectins from Edible Mushrooms

Generally, mushroom lectins show high diversity in the N-terminal sequences, followed by some conserved sequences [10,12]. In the early 90s, Kawagishi *et al.*, determined the N-terminal amino acid sequence of *Pholiota aurivella* agglutinin (PAA) [58]. The amino acid sequence analysis gave no evidence of heterogeneity in the primary structure of the first 16 N-terminal residues (YSVTTPNSVKGGTNQG) which has a high content of serine, glycine, and acidic amino acids [107].

AAL is an *Agrocybe aegerita* lectin with an amino acid composition rich in neutral nonpolar amino acids (glycine, alanine, valine) and acidic amino acids. This lectin also has a low content of methionine, arginine, lysine and histidine residues and traces of cysteine residues which is consistent with the low isoelectric point of AAL. The amino end of the native AAL contained pyroglutamyl and after treatment with pyroglutamate aminopeptidase, the sequence of the first eight N-terminal amino acids of AAL was determined to be (QGVNIYNI) [37,38,113]. Zhang *et al.* studied the N-terminal amino-acid sequence (DILMGTYGML) of *Pholiota adiposa* lectin (PAL) and they found that the sequence showed little similarity to sequences of other published Agaricales mushroom lectins, such as lectins from *Agaricus bisporus*, *Agrocybe aegerita*, *Coprinopsis cinerea*, *Flammulina velutipes*, *Laccaria bicolor*, *Marasmius oreades*, *Pholiota aurivella*, and *Pleurotus cornucopiae* [82].

Thakur *et al.* reported comparatively longer N-terminal sequences from *Ganoderma lucidum* lectin which does not indicate similarity to any known lectin. The first 20 residues of the lectin have been reported as QFIYNGKFNWLNYALNETIT [10,12]. *Hericium erinaceum* lectin isolated by Li *et al.* possesses a distinctive N-terminal sequence AFGQLSFANLAAADF, with little resemblance to some of the published mushroom lectins [103]. Even more recently, Wang *et al.* presented N-terminal amino acid sequence of the *Paxillus involutus* lectin (CTCAVFLNNTTVKS), which has a low level of similarity to previously reported mushroom lectin sequences [106].

Fungal immunomodulatory proteins (FIP) are also well known for their similar bio-functional activities with that of lectins. Both types of proteins are able to agglutinate RBCs and bind to cell surface sugar moieties. There are some reports on the full amino acid sequences of such FIP and lectins from edible mushrooms [31]. To mention a few, they are *Aleuria aurantia* lectin, *Hygrophorus russula* lectin, LZ-8 and LZ-9 from *Ganoderma lucidum*, FIP-*fve* from *Flammulina velutipes* and FIP-*gts* from *Ganoderma tsugae* [51,61,62,63]. By knowing the full sequence of a protein, one can study the importance of a specific region by deletion analysis of amino acids in that domain. For example, after deletion analysis of the N-terminal amphipathic α-helix domain of FIP-*gts*, Lin *et al.* (1997) identified that the sequence of first 10 amino acids is responsible for inducing the immunomodulatory activity and the sequence of first 13 amino acids is responsible for dimerization of this protein [114].

A list of N-terminal sequences of lectins from different edible mushrooms is presented in Table 2 and full amino acid sequences for some lectins and related FIP from edible mushrooms in Table 3. These represent the sequence information available about these proteins. A comparison of N-terminal sequences reveals the differences among the various isolated lectins although it does not exclude the possibility of substantial sequence homology in the remaining part of the sequences.

**Table 3:** Full amino acid sequences of some lectins and related fungal immunomodulatory proteins (FIP) from edible mushrooms.

Lectins/FIP	Complete Amino Acid Sequences	References
*Aleuria aurantia* lectin	1-PTEFLYTSKIAAISWAATGGRQQRVYFQDLNGKIREAQRGGDNPWTGGSSQNVIGEAKLFSPLAAVTWKSAQGIQIRVYCVNKDNILSEFVYDGSKWITGQLGSVGVKVGSNSKLAALQWGGSESAPPNIRVYYQKSNGSGSSIHEYVWSGKWTAGASFGSDVPGDGIGATAIGPGRIRIYYQATINKIREHQQDSNSWYVGGFSASASAGVSIAAISWGSTPNIRVYWQKGREELYEAAYGGSWNTPGQIKDASRPTPSLPDTFIAANSSGNIDISVFFQASGVSLQQWQWISGKGWSIGAVVPTGTPAGW-312	Fukumori [100]
FIP-*fve*	1-MSATSLTFQLAYLVKKIDFDYTPNWGRGTPSSYIDNLTFPKVLTDKKYSYRVVVNGSDLGVESNFAVTPSGGQTINFLQYNKGYGVADTKTIQVFVVIPDTGNSEEYIIAEWKKT-115	Bastiaan-Net [115]
FIP- *gts*	1-SDTALIFRLAWDVKKLSFDYTPNWGRGNPNNFIDTVTFPKVLTDKAYTYRVAVSGRNLGVKPSYAVESDGSQKVNFLEYNSGYGIADTNTIQVFVVDPDTNNDFIIAQWN-110	Lin [114]
*Hygrophorus russula* lectin (HRL)	1-TIGTAKPILAQTAIVGGPSVPFDDAREVASWPAKLEIAQDFPITGITVRHGQIINNLTIIYRTVNGNSATVSHGGDSGGIVDKVALNENEIITSVQGRAGQHRSYNRPYLDSISFTILDTKTLVTRTTNIFGNGDGTNQGDPFQVAQPYAFAGATYTDGQTGVAGLSFFKVITNA-175	Suzuki [61]
LZ-8	1-MSDTALIPRLAWDVKKLSFDYPTNWGRGNPNNFIDTVTFPKVLTDKAYTYRVAVSGRNLGVKPSYAVESDGSQKVNFLEYNSGYGIADTNTIQVFVVDPDTNNDFIIAQWN-111	Bastiaan-Net [115]
LZ-9	1-MSDTALIPRLAWEIKKLAFDYPTNWGRGNPSSYIDTVTFPQVLTGKEYTYRVAVSGKDLGVRPSYAVESDGSQKVNFLEYNAGYGIADKNTIQVYVIDPDTGNDFIIAQWN-111	Bastiaan-Net [115]

## 6. Conclusions

Edible mushrooms are not only known for their flavors and culinary features. Evidence has been accumulating that mushrooms have nutritional as well as medicinal value. They have also been universally acknowledged as valuable sources of biologically important compounds having many potential applications in health sciences [116]. Other than polysaccharides, mushrooms produce a large number of pharmacologically active proteins, including fungal immunomodulatory proteins (FIP), ribosome inactivating proteins (RIP), antibacterial/antifungal proteins, lectins, ribonucleases, laccases and other proteins [3,116]. They are indeed good sources of novel lectins with unique specificity and potential for biomedical and biotechnological applications. Among the various lectins reported in the literature, lectins from edible mushrooms still have a juvenile status in terms of their structural and functional characterization. There are reports on lectins from edible mushrooms and their applications based on immunomodulating, antiproliferative, and antiviral/antimicrobial activities. It is also obvious that there is a need for further studies on the structural characterization of these lectins in terms of amino acid sequences, X-ray crystallography studies, and proteomics, as well as to explore various aspects to elucidate their structure–function relationships.

More information on N-terminal sequences and genomic analyses on these lectins would be helpful in appraising these important biomolecules. Hence, many food-producing industries and pharmaceutical companies need to focus on cultivating and harboring lectins from wild edible mushrooms. Although there are reports on the biological properties of lectins from edible mushrooms, very few have shown how these molecules actually play their role in the host organisms. This area still remains obscure. It is hoped that these lectins from edible mushrooms can be developed into clinically useful drugs and may be useful in designing new therapeutic drugs for many human diseases.
